# Impalpable Testis: Evaluation of Diagnostic and Treatment Procedures and Our Treatment Protocol

**DOI:** 10.1155/2018/3143412

**Published:** 2018-07-17

**Authors:** Ivana Fratrić, Dragan Šarac, Jelena Antić, Marina Đermanov, Radoica Jokić

**Affiliations:** ^1^Institute for Children and Youth Healthcare of Vojvodina, Clinic for Pediatric Surgery, Hajduk Veljkova 10, 21000 Novi Sad, Serbia; ^2^University of Novi Sad, Faculty of Medicine, Department of Surgery, Hajduk Veljkova 3, 21137 Novi Sad, Serbia

## Abstract

**Introduction:**

The aim of this study is to present our treatment protocol for impalpable testis.

**Material and Methods:**

In a retrospective study we analyzed clinical data including diagnostic procedures, intraoperative findings, final diagnosis, treatment modality, and outcome of patients with impalpable testis who underwent surgery from January 2010 until December 2015.

**Results:**

Ninety-one patients were admitted under the diagnosis of impalpable testis. In 39 patients ultrasound detected testis in the inguinal canal and orchidopexy was done. In 25 patients (48.08%) laparoscopy showed the entrance of the spermatic cord into the inguinal canal. Open exploration of the inguinal canal was done, testicular remnant removed, and appropriate testicular prosthesis implanted. Twenty patients (20/52) underwent orchidopexy of the abdominal testis (46.51%), 4 of which underwent Fowler-Stevens procedure in two stages, and in 16 patients deliberation of the testis and spermatic cord was sufficient to place the testis into the scrotum.

**Conclusions:**

Excision of the testicular nubbin is highly recommendable, as well as implantation of the testicular prosthesis at the time of orchiectomy.

## 1. Introduction

Cryptorchidism or undescended testis is one of the most common congenital malformations in male neonates and is related to a multifactorial process [1]. Incidence varies and depends on gestational age, affecting 1.0-4.6% of full-term and 1.1-45.0% of preterm neonates.

Despite spontaneous descent within the first months of life, nearly 1.0% of all male infants still have undescended testes at 1 year of age [2]. The most useful classification of undescended testes is into palpable and nonpalpable testes, and clinical management is decided by the presence and location of the testes. Approximately 80% of all undescended testes are palpable [3]. A testis may be impalpable because of intra-uterine regression (vanishing testis), agenesis (true monorchia), intra-abdominal location, inguinal location with a different grade of dysplasia or atrophy, or a position of the testis outside its normal route of descent [4]. It is essential to make a timely and correct diagnosis, to ensure that the patient receives proper treatment at the appropriate time [5]. Once the diagnosis of impalpable testis has been made, an appropriate treatment protocol should be followed. An optimal treatment protocol is, however, a matter of debate. We would like to present our current treatment protocol.

## 2. Patients and Method

From January 2010 to December 2015, 493 patients with undescended testis underwent surgery at the Institute for Children and Youth Healthcare of Vojvodina. Ninety-one patients (18.5%) had impalpable testes. A retrospective review was conducted. Data that we collected were diagnostic procedures used for a confirmation of a diagnosis of impalpable testis. Intraoperative findings, final diagnosis, treatment modality, and outcome were noted for every patient. The study was approved by Institutional Ethical Board.

## 3. Operative Procedure

**Figure 1:**
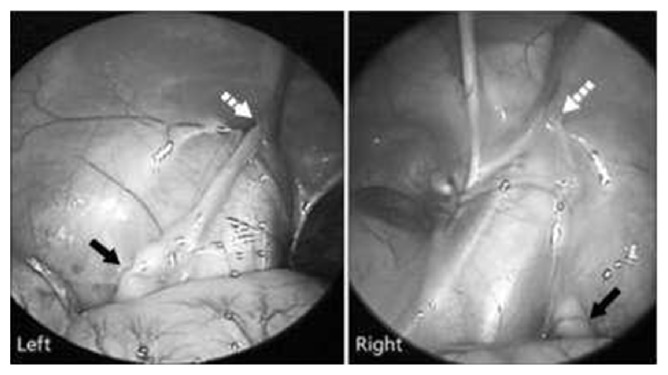
Laparoscopic approach showing both intra-abdominal testes proximal to the internal inguinal ring. The straight arrow is the testis and the dotted arrow is the internal inguinal ring.

All patients with impalpable testis and negative ultrasound underwent abdominal laparoscopy. Open Hasson technique with a subumbilical incision was used to gain entry to the abdomen for laparoscopy, for insertion of the first port and creation of pneumoperitoneum. If the testis was identified intra-abdominally (Figure 1), two 3 or 5 mm working ports were inserted as well. Then one-stage laparoscopic or two-staged laparoscopic Fowler-Stevens orchidopexy was performed. If vas and testicular vessels entered into the deep inguinal ring, a standard inguinal exploration and orchidopexy were then performed simultaneously.

**Figure 2:**
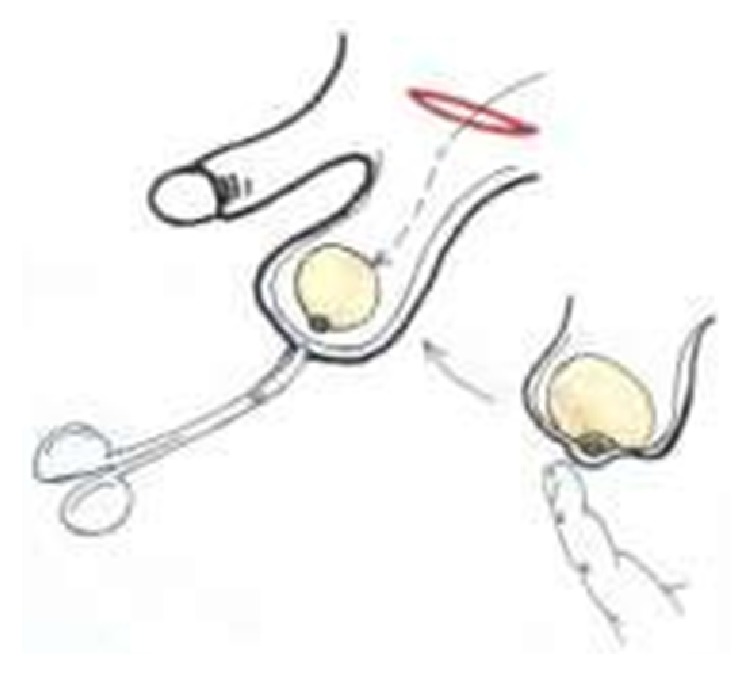
Implantation of the testicular prosthesis.

In the case when no testis was identified or testicular remnant was present during inguinal exploration, the remnant was removed and an adequate testicular prosthesis was implanted during the same operation (Figure 2). The presence of blind-ending spermatic vessels suggests the absence of testis, allowing for termination of the exploration and leaving us with just a need for prosthesis implantation [6].

Statistical analysis was performed using the SPSS program. Fisher exact test was used to compare the categorical data. P value less than 0.05 was considered statistically significant.

## 4. Results

Ninety-one patients were admitted under the diagnosis of impalpable testis. All patients underwent ultrasound examination that revealed an inguinal testis in thirty-nine patients. All of those patients underwent inguinal orchiopexy.

In 52 patients, ultrasound did not detect testis in the inguinal canal. Those patients were treated for impalpable testis by laparoscopy. Patients were 1-17 years old (average 3.83). Forty-three patients (82.69%) had unilateral impalpable testis and eighteen of these patients had right-sided (41.86%) and 25 had left-sided impalpable testis (58.14%). Nine patients (17.31%) had bilateral impalpable testes with either positive or unclear stimulation test. Twenty-five patients (48.08%) underwent laparoscopic exploration that confirmed the entrance of the spermatic cord into the inguinal canal. Open exploration of the inguinal canal was done, testicular remnant removed, and appropriate testicular prosthesis implanted (18 testicular prostheses No I, 5 prostheses No II, and 2 prostheses No III; Table 1).

Twenty patients (20/52) underwent orchidopexy of the abdominal testis (46.51%), 4 of which underwent Fowler-Stevens procedure in two stages, and in 16 patients deliberation of the testis and spermatic cord was sufficient to place the testis into the scrotum. One of the patients with bilateral abdominal testes was operated in a single stage laparoscopy procedure, but required a subsequent open reorchidopexy on one side.  Table 2 presents the association of the need for inguinal exploration and orchidopexy in children with unilateral or bilateral impalpable testes.

In 7 patients (7/52) laparoscopy confirmed the entrance of the spermatic cord into the inguinal canal, where in three patients we found hypoplastic testis that we decided to leave in place, and in other 4 patients the remnant was removed, but prosthesis was not implanted due to a lack of either the appropriate size or parental consent.

**Figure 3:**
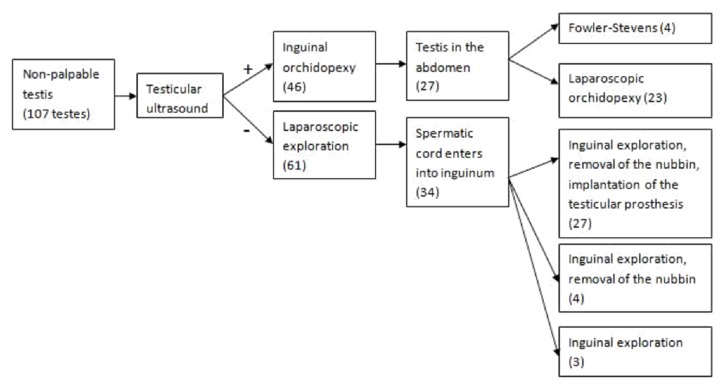
Treatment protocol.

Our treatment protocol and results are presented in Figure 3.

## 5. Discussion

At the Institute for Child and Youth Healthcare of Vojvodina, a child with undescended testis is followed from birth to the age of 9 months, during which time clinical examination is performed at birth, at the age of 6 months, and at the age of 9 months. Hypertrophic contralateral testis could be correlated with absent or atrophic testis [6, 7]. Nevertheless, this does not preclude surgical exploration of the abdomen and/or inguinal canal, since the sign of compensatory hypertrophy is not specific enough [8]. If testis is impalpable, ultrasound examination is always performed. In expert hands, ultrasonography has a high positive predictive value for inguinal located testes, such as 91% with 78% sensitivity [9]. When ultrasound is done by a pediatric radiologist, and testis is located in an inguinal location, a primary inguinal exploration can be considered, preventing an unnecessary diagnostic laparoscopy [10]. Laparoscopy has become the new “gold standard” diagnostic method for impalpable testis. In addition to being a diagnostic method, it has been an option for treating this condition [6].

An important step in surgery is a thorough clinical examination once the boy is under general anesthesia, as a previously impalpable testis might be identified during this examination and subsequently the planned laparoscopic surgical approach can be changed to standard inguinal orchidopexy. However, even under anesthesia it can be difficult to palpate a testicular nubbin in the scrotum, especially in an obese child [10]. Some studies show that although a nonpalpable testis was diagnosed by a pediatric surgeon and urologist, an inguinal testis was found in 21–85% of the patients during surgery [4]. This kind of examination is not a standard part of our treatment protocol due to organizational issues. Namely, both a child and an operating theater are prepared for either conventional or laparoscopic surgery. It is, however, one of the things that we are planning to change in our protocol.

The standard management of impalpable testes is surgical, which could be challenging and controversial. Orchidopexy is indicated if the testis is not located in the scrotum after 6 months as it improves fertility rates and allows close monitoring for the development of possible testicular masses [11]. Therefore we tend to perform surgery early enough, but we also wait a little bit longer compared to the timing of treatment for patients with palpable undescended testis (18, instead of 12 months), in order for the scrotal sac to allow us to place a testicular prosthesis. According to our treatment protocol, we start with laparoscopic examination. It is the easiest and most accurate way to locate an intraabdominal testis [12]. Subsequent testicular nubbin removal or orchidolysis and orchidopexy can be carried out using the same approach to achieve the therapeutic aims [12]. Some surgeons tend to start with inguinal surgical exploration, with possible laparoscopy during the procedure [13]. After years of practice it might be advisable to consider inguinal approach, together with examination under anesthesia as a first line of treatment. In that way we would avoid laparoscopies that might be unnecessary and time consuming, especially in obese children.

Another controversial issue is the need for removal of testicular nubbin. Nubbin is a congenital condition in which no normal testicular tissue can be identified following exploration for a clinically impalpable testis [14]. This includes the presence of a testicular remnant, nodule, or strand of testicular/paratesticular tissue at the end of the spermatic cord. The main reason for the debate over the management of patients with testicular regression syndrome (TRS) is the variable incidence of viable germ cells reported in different studies: between 0 and 16% [15–21]. The concern with the presence of seminiferous tubules is that there is the potential for viable germ cells to be present, although they may have not been identified specifically on histological analysis. This variance may be secondary to the different histopathological analysis practices in the various studies. There is only one case of intratubular germ cell neoplasia in a testicular remnant reported in the literature and this was not immunohistochemically supported [16]. Although this malignancy risk remains controversial we believe that it should be recommended that these are excised as one in 10 specimens has germ cells present and one in four has seminiferous tubules.

The absence of a testis from the scrotal sac represents a psychologically traumatic experience in males of any age from childhood to the elderly [22, 23]. Therefore, surgery for testicular prosthesis implantation is a solution that minimizes the psychological consequences of the absence of the testicle in the scrotum, providing similarity in size, weight, and appearance of natural testicle.

The timing of insertion of a testicular prosthesis in a child is not unequivocal. The psychological impact of an absent testicle in a child or adolescent is a good reason to consider implantation at the time of the initial surgery for a cryptorchid testis. The problem is that this may necessitate further surgery to insert a larger prosthesis when the child gets older. An alternative strategy is to delay the placement of the definitive prosthesis until the child reaches adolescence. If a child is content with the size of the initially implanted prosthesis, further surgery can be avoided. The underdeveloped scrotum that accompanies an undescended testicle may fail to accommodate the desired sized testicular prosthesis in adolescence.

## 6. Conclusion

We believe that recent improvements in our protocol, including introducing the additional clinical examination in anesthesia and switch to initial inguinal approach, hold promise of improved outcomes. Even when inguinal exploration is being done, it is advisable to be ready to perform laparoscopic exploration of the abdomen in case of negative finding in the inguinal canal. Excision of the testicular nubbin is in our experience highly recommendable, as well as implantation of the testicular prosthesis at the time of orchiectomy.

## Figures and Tables

**Table 1:** The size of the implanted testicular prosthesis.

**The size of the implanted** **testicular prosthesis**	**Number of patients**	**Percentage of patients [**%**]**
**No I**	18	72
**No II**	5	20
**No III**	2	8
**Total**	25	100

**Table 2:** The association of the need for inguinal exploration and orchidopexy in children with unilateral or bilateral impalpable testis (based on the number of procedures performed).

		**Unilateral** **impalpable** **testis**	**Bilateral** **impalpable** **testis**	**p value**
		**n = 43**	**n = 9**	
**Inguinal exploration**	Required	25	7	0.45
	Not required	18	2	
**Orchidopexy**	Performed	20	1	0.07
	Not performed	23	8	

## Data Availability

Data are available upon request by the corresponding author through the provided e-mail.
