# Corrigendum to “Impalpable Testis: Evaluation of Diagnostic and Treatment Procedures and Our Treatment Protocol”

**DOI:** 10.1155/2021/6890257

**Published:** 2021-01-28

**Authors:** Ivana Fratrić, Dragan Šarac, Jelena Antić, Marina Đermanov, Radoica Jokić

**Affiliations:** ^1^University of Novi Sad, Faculty of Medicine, Department of Surgery, Hajduk Veljkova 3, 21137 Novi Sad, Serbia; ^2^Institute for Children and Youth Healthcare of Vojvodina, Clinic for Pediatric Surgery, Hajduk Veljkova 10, 21000 Novi Sad, Serbia

The article titled “Impalpable Testis: Evaluation of Diagnostic and Treatment Procedures and Our Treatment Protocol” [1] was found to contain a substantial amount of material, without citation, from previously published articles. This has been corrected in the revised version shown below:

(1) The complete article has been updated
(2) Three references were added [2–4]:
  - Ximena Sepúlveda, Pedro-José López Egaña, “Current management of non-palpable testes: a literature review and clinical results,” Translational Pediatrics, 5(4), 2016 doi:10.21037/tp.2016.10.06
  - Bodiwala D, Summerton DJ, Terry TR. Testicular prostheses: development and modern usage. Ann R Coll Surg Engl. 2007 May; 89(4):349-53. doi:10.1308/003588407X183463
  - Radmayr C, Dogan HS, Hoebeke P, et al. Management of undescended testes: European Association of Urology/European Society for Paediatric Urology Guidelines [published correction appears in J Pediatr Urol. 2017 Apr; 13(2):239]. *J Pediatr Urol*. 2016; 12(6):335-343. doi:10.1016/j.jpurol.2016.07.014

**Abstract**

*Introduction*. Cryptorchidism is one of the most frequent congenital malformations in male newborn. The aim of this paper is to present current literature recommendations and our treatment protocol of impalpable testis based on our experience. *Material and methods*. This is a retrospective study where we analyzed clinical documents from January 2010 to December 2015. We reviewed diagnostic procedures, intraoperative findings with closing diagnosis, and all treatment modalities for patients with impalpable testis. We also investigated current literature regarding this topic. *Results*. Ninety-one patients were admitted under the diagnosis of impalpable testis. In 39 patients, ultrasound detected testis in the inguinal canal and standard open orchidopexy was done. In 25 patients, laparoscopy showed (48.08%) the entrance of the spermatic cord into the inguinal canal, then open exploration of canal was performed, testicular remnant removed, and simultaneously appropriate testicular prosthesis was implanted. Next, twenty patients underwent orchiopexy of the abdominal testis. Four of them underwent the Fowler-Stevens procedure in two stages, and in 16 patients, deliberation of the testis was sufficient to place the testis into the scrotum during the same operation. *Conclusions*. Improvements in therapeutical protocol, with additional clinical examination in anesthesia, switch to initial inguinal approach and laparoscopic exploration of the abdomen in case of negative finding in the inguinal canal could improve outcome for these patients. If testicular nubbin is found during exploration, its excision is highly recommendable, as well as implantation of the testicular prosthesis at the same time during the surgical orchiectomy.

## 1. Introduction

Among all congenital malformations in boys at the time of birth, cryptorchidism is the most common [1, 2]. Although the exact etiology of this condition is unknown, it is believed to be the result of multiple factors [1, 2]. It affects preterm neonates much more than full-term boys (1.1 -45.0% vs. 1.0-4.6%) [2, 3]. The definitive diagnosis of undescended testis is made after six months of age due to the postnatal spontaneous testicular descent. However, nearly 1% of 1-year-old boys have the diagnosis of cryptorchidism [2, 3]. The most useful clinical classification is into palpable and nonpalpable testes. Almost 80% of undescended testes are palpable [4]. When considering anatomical location, nonpalpable testis generally could be absent due to prenatal regression (vanishing testis), agenesis (true monorchia), and located in the abdomen or in inguinal canal. Furthermore, testis could be located anywhere outside its normal path of descent when it is called ectopic testis [5, 6]. It is crucial for appropriate therapy to make precise diagnosis at a timely manner (6 to 12 months) [6, 7]. Once the diagnosis of impalpable testis has been made, a suitable treatment protocol should be strictly followed. An optimal treatment protocol is however a matter of debate in the current literature. In this paper, we would like to present our latest treatment protocol.

## 2. Patients and Method

From January 2010 to December 2015, 493 patients with undescended testis underwent surgery at the Institute for Child and Youth Healthcare of Vojvodina. Ninety-one patients (18.5%) had impalpable testes. A retrospective review was conducted. Data that we collected were diagnostic procedures used for a confirmation of a diagnosis of impalpable testis. Intraoperative findings, final diagnosis, treatment modality, and outcome were noted for every patient. This study has the approval of the Institutional Ethical Board.

### 2.1. Operative Procedure

Patients with impalpable testis and negative ultrasound underwent abdominal laparoscopy. Open Hasson technique with infraumbilical incision was used to gain entry to the abdomen for laparoscopy, for insertion of the first port, and creation of pneumoperitoneum. If the testis is found intraabdominally, the two additional ports were inserted as well. They are so-called working ports. According to the finding, the surgeon could decide either to finish the laparoscopic procedure as one or two-stage Fowler-Stephens orchidopexy. In case with vas deferens and gonadal vessels entering the deep inguinal ring which means that testis or testicular remnant is present in the inguinal canal, standard inguinal exploration and eventually open orchidopexy were performed.

In the case when no testis was identified or testicular remnant was present during inguinal exploration, the remnant was removed and an adequate testicular prosthesis was implanted simultaneously (Figure 1). The finding of spermatic vessels ending blind suggests the lack of testis, allowing the end of the exploration and enables implantation of prosthesis as well [8].

### 2.2. Statistical Analysis

We used the SPSS program for statistical analysis. Categorical data were compared using the Fisher exact test. While discussing results, *P* value that was 0.05 or less was used for the determination of statistical significance.

## 3. Results

Ninety-one patients were admitted under the diagnosis of impalpable testis. All patients underwent an ultrasound examination that revealed an inguinal testis in thirty-nine patients. All of those patients underwent inguinal orchiopexy.

In 52 patients, ultrasound did not detect testis in the inguinal canal. Those patients were treated for impalpable testis by laparoscopy. Patients were 1-17 years old (average 3.83). Forty-three patients (82.69%) had unilateral impalpable testis, eighteen of these patients had right-sided (41.86%), and 25 had left-sided impalpable testis (58.14%). Nine patients (17.31%) had bilateral impalpable testes with either positive or unclear stimulation test. Twenty-five patients (48.08%) underwent laparoscopic exploration that confirmed the entrance of the spermatic cord into the inguinal canal, followed by open exploration, removal of testicular remnant and implantation of appropriate testicular prosthesis (18 testicular prostheses No I, 5 prostheses No II, and 2 prostheses No III—Table 1).

Twenty patients (20/52) underwent orchidopexy of the abdominal testis (46.51%), 4 of which underwent Fowler-Stevens procedure in two stages, and in 16 patients, deliberation of the testis and spermatic cord was sufficient to place the testis into the scrotum in one operation. One of the patients with bilateral abdominal testes was operated in a single-stage laparoscopy procedure bilaterally, but required a subsequent open reorchidopexy on one side.  Table 2 presents procedures performed in patients with impalpable testis

In 7 patients (7/52), laparoscopy confirmed the entrance of the spermatic cord into the inguinal canal, where in three patients we found hypoplastic testis that we decided to leave in place, and in the other 4 patients, the remnant was removed, but prosthesis was not implanted either due to a lack of the appropriate size or parental consent. Therefore, in these seven patients we did not place the prosthesis during the same operation.

Our treatment protocol and results are presented in Figure 2.

## 4. Discussion

At the Institute for Child and Youth Healthcare of Vojvodina, a child with undescended testis is followed from birth. During that time, the clinical exam is performed minimally three times: days after birth, at 6 and 9 months of age. The finding of enlarged contralateral testis could suggest absent or atrophic testis ipsilaterally [6, 8, 9]. However, that should not withdraw the patient from surgical exploration of the abdomen and/or inguinal canal [6, 10]. If testis is clinically impalpable, an ultrasound examination is always performed. Ultrasound can confirm the presence of almost 80% of all testes located in the inguinal canal with an accuracy of more than 90% [6, 11]. The high precision suggests that inguinal exploration and orchidopexy could be performed without diagnostic laparoscopy [6, 12]. In any case, diagnostic and therapeutic laparoscopy has become a modern useful asset or a new “gold standard” for treating patients with impalpable testis [6, 8].

Surgeons should always use a chance to perform one more clinical examination during general anesthesia [2]. Sometimes, the previously impalpable testis is found and the planned laparoscopic operation could be cancelled. Then, orchidopexy is performed using a classical open approach (standard inguinal orchidopexy). Literature suggests that a wide range of around 20% to 85% of inguinal testis could be found during surgery in patients previously diagnosed with nonpalpable testis [5, 6]. This kind of examination under general anesthesia is not a standard part of our treatment protocol due to organizational issues. Namely, both a child and an operating theater are prepared for either conventional or laparoscopic surgery. It is however one of the things that we are planning to change in our protocol.

The standard management of impalpable testes is primarily surgical. According to the newest literature, orchidopexy should be performed by the age of 6 months as it may improve the fertility potential and prevent possible tumor formation [6, 13]. Therefore, we tend to perform surgery early enough, but we also wait a little bit longer compared to the timing of treatment for patients with palpable undescended testis (18, instead of 12 months). The reason for this short delay is enabling the scrotal sac to grow enough to receive a testicular prosthesis.

According to our treatment protocol, we start with laparoscopic examination which allows accurate diagnosis for intraabdominal testis [14], subsequent testicular nubbin removal, or one or two-stage Fowler-Stevens procedure. On contrary, there are some surgeons who prefer open inguinal exploration and after that planned laparoscopy for undiscovered testis [2, 15]. After years of practice, it might be advisable to consider the inguinal approach, together with examination under anesthesia as a first line of treatment. In that way, we would avoid laparoscopies that might be unnecessary and time-consuming, especially in obese children. Another controversial issue is the need for obligatory removal of testicular nubbin [16]. The treatment of patients with testicular regression syndrome (TRS) is controversial because 0 to 16% of them have viable germ cells [17-23]. If left in place, testicular remnant could become malignant according to one case reported in literature [24]. Although scientific conclusion should not be derived from one study alone, we also believe that testicular remnant should be excised as in 25% of cases seminiferous tubules are found during histological analysis and in 10% germ cells are found as well.

Having an undescended testis is for many boys and even older men psychological issue [25-27] that could be partially solved by implantation of testicular prosthesis. As prosthesis does not grow with the child, it is of great importance to plan the correct timing for surgery [27]. If performed too early, it may lead to another surgery for a larger prosthesis. On the other hand, it prevents possible problems with undeveloped scrotum in adolescence which might not be suitable for larger testicular prosthesis insertion [27].

## 5. Conclusion

We believe that recent improvements in our protocol, including introducing the additional clinical examination in anesthesia and switch to an initial inguinal approach, hold promise of improved outcomes. Even when inguinal exploration is being done, it is advisable to be ready to perform laparoscopic exploration of the abdomen in case of negative finding in the inguinal canal. Excision of the testicular nubbin is in our experience highly recommendable, as well as implantation of the testicular prosthesis at the time of orchiectomy.

**References**

1. H. E. Virtanen, R. Bjerknes, D. Cortes, N. Jørgensen, E. Rajpert-de Meyts, A. V. Thorsson, J. Thorup, and K. M. Main, “Cryptorchidism: classification, prevalence and long-term consequences,” *Acta Paediatrica*, vol. 96, pp, 611–616, 2007, doi:10.1111/j.1651-2227.2007.00241.x.

2. C. Radmayr, H. S. Dogan, P. Hoebeke, R. Kocvara, R. Nijman, S. Silay, R. Stein, S. Undre, and S. Tekgul, “Management of undescended testes: European Association of Urology/European Society for Paediatric Urology Guidelines,” *Journal of Pediatric Urology*, vol. 12, no. 6, pp. 335–343, 2016. doi:10.1016/j.jpurol.2016.07.014.

3. K. Sijstermans, W. W. Hack, R. W. Meijer, and L. M. van der Voort-Doedens, “The frequency of undescended testis from birth to adulthood: a review,” *International Journal of Andrology*, vol. 31, pp. 1–11, 2008, doi:10.1111/j.1365-2605.2007.00770.x.

4. C. Kollin and E. M. Ritzen, “Cryptorchidism: a clinical perspective,” *Pediatric Endocrinology Reviews*, vol. 11, pp. 240–250, 2014.

5. D. A. Diamond and A. A. Caldamone, “The value of laparoscopy for 106 impalpable testes relative to clinical presentation,” *The Journal of Urology*, vol. 148, pp. 632–634, 1992, doi:10.1016/S0022-5347(17)36675-2.

6. X. Sepúlveda and P.-J. L. Egaña, “Current management of non-palpable testes: a literature review and clinical results,” *Translational Pediatrics*, vol. 5, no. 4, 2017, doi:10.21037/tp.2016.10.06.

7. I. R. Budianto, H. L. Tan, Y. Kinoshita, R. P. Tamba, S. Leiri, and T. Taguchi, “Role of laparoscopy and ultrasound in the management of "impalpable testis" in children,” *Asian Journal of Surgery*, vol. 37, pp. 200–204, 2014, doi:10.1016/j.asjsur.2014.01.013.

8. S. G. Docimo, R. I. Silver, and W. Cromie, “The undescended testicle: diagnosis and management,” *American Family Physician*, vol. 62, no. 9, pp. 2037–2044, 2000.

9. F. T. Denes, F. J. Saito, F. A. Silva, A. M. Giron, M. Machado, and M. Srougi, “Laparoscopic diagnosis and treatment of nonpalpable testis,” *International Brazilian Journal of Urology*, vol. 34, pp. 329–335, 2008, doi:10.1590/S1677-55382008000300010.

10. R. S. Hurwitz and J. S. Kaptein, “How well does contralateral testis hypertrophy predict the absence of the nonpalpable testis?” *The Journal of Urology*, vol. 165, pp. 588–592, 2001, doi:10.1097/00005392-200102000-00077.

11. A. Vos, A. Meij-de Vries, A. Smets, J. Verbeke, H. Heij, and A. van der Steeg, “The value of ultrasonography in boys with a non-palpable testis,” *Journal of Pediatric Surgery*, vol. 49, pp. 1153–1155, 2014, doi:10.1016/j.jpedsurg.2013.09.011.

12. S. M. P. Nijs, S. W. Eijsbouts, G. C. Madern, P. M. M. Leyman, M. H. Lequin, and F. W. J. Hazebroek, “Nonpalpable testes: is there a relationship between ultrasonographic and operative findings?” *Pediatric Radiology*, vol. 37, pp. 374–379, 2007, doi:10.1007/s00247-007-0425-1.

13. K. H. Park, J. H. Lee, J. J. Han, S. D. Lee, and S. Y. Song, “Histological evidences suggest recommending orchiopexy within the first year of life for children with unilateral inguinal cryptorchid testis,” *International Journal of Urology*, vol. 14, pp. 616–621, 2007, doi:10.1111/j.1442-2042.2007.01788.x.

14. K. W. E. Chan, K. H. Lee, H. Y. V. Wong, S. Y. B. Tsui, Y. S. Wong, K. Y. K. Pang, J. W. C. Mou, and Y. H. Tam, “Use of laparoscopy as the initial surgical approach of impalpable testes: 10-year experience,” *World Journal of Clinical Pediatrics*, vol. 4, pp. 155–159, 2015, doi:10.5409/wjcp.v4.i4.155.

15. V. V. Chandrasekharam, “Laparoscopy vs inguinal exploration for nonpalpable undescended testis,” *The Indian Journal of Pediatrics*, vol. 72, pp. 1021–1023, 2005, doi:10.1007/BF02724403.

16. R. M. Nataraja, C. M. Asher, R. Nash, and F. L. Murphy, “Is routine excision of testicular remnants in testicular regression syndrome indicated?” *Journal of Pediatric Urology*, vol. 11, pp. 151.e1–151.e5, 2015, doi:10.1016/j.jpurol.2015.01.018.

17. D. Storm, T. Redden, M. Aguiar, M. Wilkerson, G. Jordan, and J. Sumfest, “Histologic evaluation of the testicular remnant associated with the vanishing testes syndrome: is surgical management necessary?” *Urology*, vol. 70, pp. 1204–1206, 2007, doi:10.1016/j.urology.2007.08.020.

18. T. A. Rozanski, K. J. Wojno, and D. A. Bloom, “The remnant orchiectomy,” *Journal of Urologys*, vol. 155, pp. 712–714, 1996, doi:10.1016/S0022-5347(01)66507-8.

19. H. Law, I. Mushtaq, K. Wingrove, M. Malone, and N.J. Sebire, “Histopathological features of testicular regression syndrome: relation to patient age and implications for management,” *Fetal and Pediatric Pathology*, vol. 25, 2009.

20. P. K. Hegarty, I. Mushtaq, and N. J. Sebire, “Natural history of testicular regression syndrome and consequences for clinical management,” *Journal of Pediatric Urology*, vol. 3, pp. 206-208, 2007, doi:10.1016/j.jpurol.2006.08.007.

21. N. Ueda, Y. Shiroyanagi, H. Suzuki, W. J. Kim, Y. Yamazaki, and Y. Tanaka, “The value of finding a closed internal ring on laparoscopy in unilateral nonpalpable testis,” *Journal of Pediatric Surgery*, vol. 48 pp. 542–546, 2013, doi:10.1016/j.jpedsurg.2012.09.032.

22. J. F. Renzulli, R. Shetty, S. Mangray, K. R. Anderson, R. M. Weiss, and A. A. Caldamone, “Clinical and histological significance of the testicular remnant found on inguinal exploration after diagnostic laparoscopy in the absence of a patent processus vaginalis,” *The Journal of Urology*, vol. 174, pp. 1584–1586, 2005, doi:10.1097/01.ju.0000179541.92934.17.

23. H. Emir, B. Ayık, M. Eliçevik, C. Büyükünal, N. Danişmend, S. Dervişoğlu, and Y. Söylet, “Histological evaluation of the testicular nubbins in patients with nonpalpable testis: assessment of etiology and surgical approach,” *Pediatric Surgery International*, vol. 23, pp. 41–44, 2007.

24. T. A. Rozanski, K. J. Wojno, D. A. Bloom, “The remnant orchiectomy,” *Journal of Urology*, vol. 155, pp. 712–714, 1996, doi:10.1016/S0022-5347(01)66507-8.

25. J. P. Pryor, “Testicular prostheses: the patient's perception,” *British Journal of Urology*, vol. 70, pp. 420–422, 1992.

26. L. Incrocci, J. L. Bosch, and A. K. Slob, “Testicular prostheses: body image and sexual functioning,” *BJU International*, vol. 84, pp. 1043–1045, 1999.

27. D. Bodiwala, D. J. Summerton, and T. R. Terry, “Testicular prostheses: development and modern usage,” *Annals of the Royal College of Surgeons of England*, vol. 89, no. 4, pp. 349–353, 2007, doi:10.1308/003588407X183463.

## Figures and Tables

**Figure 1:**
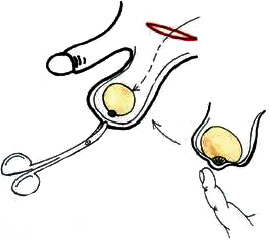
Implantation of the testicular prosthesis.

**Figure 2:**
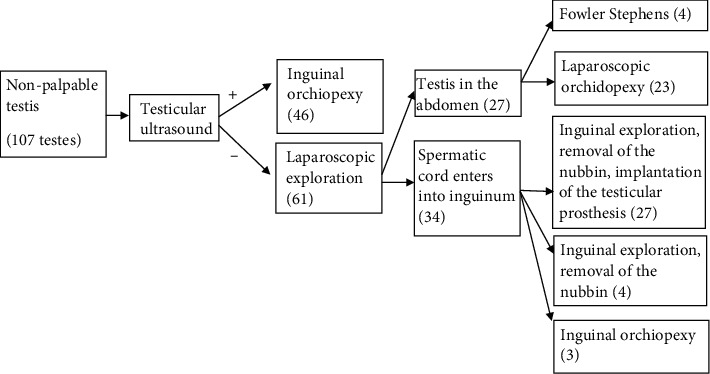
Treatment protocol.

**Table 1:** The size of the implanted testicular prosthesis.

The size of the implanted testicular prosthesis	Number of patients	Percentage of patients (%)
No I	18	72
No II	5	20
No III	2	8
Total	25	100

**Table 2:** Procedures performed in patients with impalpable testis.

		Unilateral impalpable testis *n* = 43	Bilateral impalpable testis *n* = 9	*P* value
Inguinal exploration	Required	25	7	0.45
	Not required	18	2	
Orchidopexy	Performed	20	1	0.07
	Not performed	23	8	

## Data Availability

Data are available upon request by corresponding author through provided e-mail.
